# Inflammatory pathways are central to posterior cerebrovascular artery remodelling prior to the onset of congenital hypertension

**DOI:** 10.1177/0271678X18769180

**Published:** 2018-04-13

**Authors:** Dawid Walas, Karol Nowicki-Osuch, Dominic Alibhai, Eva von Linstow Roloff, Jane Coghill, Christy Waterfall, Julian FR Paton

**Affiliations:** 1 School of Physiology, Pharmacology and Neuroscience, Biomedical Sciences, University of Bristol, Bristol, UK; 2 Faculty of Life Sciences, University of Manchester, Manchester, UK; 3 Wolfson Bioimaging Facility, School of Biochemistry, Biomedical Sciences, University of Bristol, Bristol, UK; 4 Genomics Facility, School of Biological Sciences, Bristol, UK; 5 Department of Physiology, Faculty of Medical and Health Sciences, University of Auckland, Grafton, New Zealand

**Keywords:** Cerebrovascular remodelling, transcriptomic plasticity, genetic hypertension, inflammatory pathways, fluorescent microscopy

## Abstract

Cerebral artery hypoperfusion may provide the basis for linking ischemic stroke with hypertension. Brain hypoperfusion may induce hypertension that may serve as an auto-protective mechanism to prevent ischemic stroke. We hypothesised that hypertension is caused by remodelling of the cerebral arteries, which is triggered by inflammation. We used a congenital rat model of hypertension and examined age-related changes in gene expression of the cerebral arteries using RNA sequencing. Prior to hypertension, we found changes in signalling pathways associated with the immune system and fibrosis. Validation studies using second harmonics generation microscopy revealed upregulation of collagen type I and IV in both tunica externa and media. These changes in the extracellular matrix of cerebral arteries pre-empted hypertension accounting for their increased stiffness and resistance, both potentially conducive to stroke. These data indicate that inflammatory driven cerebral artery remodelling occurs prior to the onset of hypertension and may be a trigger elevating systemic blood pressure in genetically programmed hypertension.

## Introduction

Hypertension remains the biggest ‘silent killer’ in the world^1^ with 29% of people suffering from it globally.^2^ If left untreated, hypertension presents a major risk of stroke, cardiac and renal disease.^1^ In around 95% of cases, the cause of hypertension is unknown and dubbed ‘essential’.^3^ The multifactorial nature of this syndrome has made it difficult to pinpoint the exact cause(s). Moreover, around 12–15% of hypertensive patients may be resistant to current therapies.^4^ Thus, there is a pressing need for unearthing root causes and developing new appropriate therapies.

Many patients with hypertension also have activation of the sympathetic nervous system that may occur prior to the onset of hypertension and therefore contribute to both the development and maintenance of high blood pressure (reviewed by Fisher and Paton^5^). The big and unresolved question is what triggers the increase in sympathetic nerve activity? In the early 1960s, Dickinson found a strong correlation between vertebral artery resistance and the ante-mortem blood pressure in cadavers; thus he confirmed with X-ray images showing that the vertebral arteries of hypertensive subjects were narrowed compared to their normotensive counterparts.^6–9^ However, the causality of this relationship could not be determined. The vertebral arteries provide blood to the basilar artery that perfuses the brainstem, the major region controlling sympathetic vasomotor activity that regulates blood pressure. We confirmed the correlation of heightened basilar artery resistance and narrowed vertebral and basilar arteries in the spontaneously hypertensive rat (SHR) when compared to its parental strain – the Wistar Kyoto rat (WKY).^10^ Intriguingly, the higher resistance and vertebrobasilar artery narrowing (‘remodelling’) were found in juvenile/normotensive SHR before hypertension had developed suggesting that these structural changes were not secondary to the high blood pressure.^10^ Recently, we showed that the brainstem of the SHR is hypoperfused and becomes severely hypoxic when blood pressure was equated to normotensive levels^11^ suggesting a highly limited blood flow reserve in this rat model. We also found that increasing vertebral artery resistance increased sympathetic activity (SNA).^10,12,13^ It is of interest that SNA is increased in the pre-hypertensive SHR^5,14^ but whether this is due to the remodelled vertebrobasilar arteries and a hypoperfused brainstem is not clear.

These findings led to our originally stated selfish brain hypothesis of hypertension where the brainstem of an SHR elevates SNA triggering high blood pressure in response to decreased perfusion (caused by the remodelling of the posterior cerebral arteries) and to provide adequate brain blood flow.^12^ This response is not unlike the Cushing response – increased intracranial pressure results in high cerebral vascular resistance and diminished perfusion, which activates the sympathetic nervous system to raise systemic blood pressure.^15^ However, the mechanisms for this vertebrobasilar artery remodelling remain elusive but may be highly relevant to the aetiology of human hypertension,^16^ and contribute to the susceptibility of ischemic stroke occurrence. This was upheld by the association between hypertension and posterior (but not anterior) cerebral artery infarcts.^13^ Thus, we set out to comprehensively describe the transcriptomic changes in the cerebral arteries of the SHR versus the WKY rat. This led us to build a plausible gene network that may provide clues to the mechanisms causing the remodelling both prior to, and during development of, hypertension. Finally, we performed proof of concept studies to functionally validate outputs from the gene network.

## Materials and methods

### Animals

**Figure 1.**
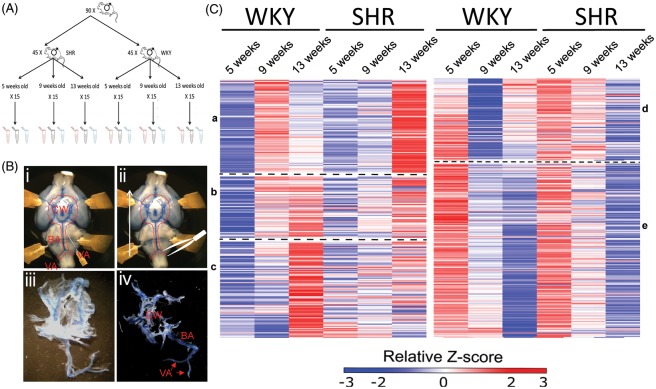
Methodology for cerebral artery transcriptomic analysis in normotensive (WKY) and spontaneously hypertensive rats (SHR) strains. (A) Summary of the animal assignment for tissue extraction. (B) Representative examples illustrating how the cerebral vessels were collected for RNA and protein extraction. (i) A 25-gauge needle was used to detach the meninges from the brain. (ii) The tissue was peeled off with watch maker forceps in the rostral direction. (iii) Collected tissue showing the meninges attached to the vessels. (iv) After stripping most of the meninges off the arteries. (C) Gross visualisation of gene transcription shifts, clustered hierarchically to WKY changes in gene expression. Each column represents a different age of either WKY rat or SHR. Each row represents the relative level of expression of a single gene. Red, high expression relative to the mean expression; blue, low expressions relative to the mean expression; white, no significant change in expression level between the sample and mean. The heat maps represent a relative longitudinal downregulation (left heat map) and upregulation (right heat map) of transcribed genes in 5 week compared to 9-week-old WKY rats. Hierarchical clustering in GENE-E grouped genes followed the same pattern of expression in aging WKY. These genes were then compared to the expression pattern in the SHR at comparable ages. The broad clusters represent similar expression patterns and are divided by the dashed lines. Compared to the development of normotensive WKY there are clear transcriptional shifts in the development of the SHR. a-c clusters of low gene expression in pre-hypertensive age. d-e clusters of high gene expression in pre-hypertensive age. CW: circle of Willis arteries; BA: basilar artery; VA, vertebral arteries.

All experiments were conducted in accordance with the UK Home Office Scientific Procedures Act 1986 under project licence PPL 30/3121, and were approved by the Local Ethical Committees on Animal Experimentation at the University of Bristol. The experiments reported here are in compliance with ARRIVE guidelines on reporting animal experiments. Animals were sourced from Harlan Laboratories. In total, 90 male rats were used for this study – 45 WKYs and 45 SHRs. The rats were delivered a week before their intended use for acclimatisation. Three age groups were used in this study – 5, 9 and 13 weeks old (Figure 1(A)). In each age group, animals were pooled into three subgroups of five, randomly assigned, animals. Each subgroup would subsequently serve as a biological replicate for the RNA sequencing (RNA-Seq) experiment. The logic of pooling animals was twofold: first to make sure that enough material was collected for the RNA-Seq platform and, second, as SHRs are greatly inbred we thought it appropriate that the ‘average’ gene expression from pooling would serve to give a better overview of the changes to gene expression. It also provided a satisfactory data quality to cost ratio.

### Tissue collection

Animals were euthanised by an overdose of sodium pentobarbital (300 mg/kg i.p.). Upon loss of the hind limb withdrawal reflex, all animals were perfused transcardially with 4℃ PBS (50 ml for 5, 80 ml for 9 and 120 ml for 13-week-old animals) to remove the blood from the cerebral circulation. Rats were then transcardially injected with Parker Quink washable ink to stain the cerebral vessels. The brain was gently but rapidly removed and the cerebral arteries (vertebral, basilar posterior communicating and the circle of Willis) peeled away from the brain (Figure 1(B)) immersed in cold PBS under a dissecting microscope. Once isolated, the vessel peel was cleaned of meninges. The cleaned arteries were snap frozen in liquid nitrogen and stored at −80℃ until RNA was extracted.

### Total RNA extraction

Total RNA was extracted using the TriReagent (Sigma–Aldrich). The protocol was modified to obtain maximum precipitation efficiency of small RNAs as described by LC Sciences (www.lcsciences.com). Briefly, at the RNA precipitation step, 1.5 ml of isopropyl alcohol was used for each millilitre of TriReagent. GlycoBlue™ Co-precipitant was added at 100 µg/ml to visualise the RNA pellet. The samples were left overnight at −20℃ to facilitate small RNA precipitation. After the incubation samples were centrifuged at 12,000 *g* for 10 min. Pellets were washed twice with 75% ethanol and pelleted again at 12,000 *g*. After air-drying the pellets for 10 min, they were solubilised in nuclease-free water. The RNA was then cleaned on Qiagen RNeasy Mini Kit for large (>200 nucleotides) RNA sequencing.

### Total RNA quality control

The quality of extracted RNA was checked on Nanodrop 1000 and Agilent 2200 TapeStation. All samples had a 260/280 ratio over 1.8. The RNA integrity number (RIN) of the total RNA was between 7.7 and 8.5. After column clean-up and removal of the small RNAs, the RIN of all samples was between 8.1 and 9.3.

### RNA sequencing

Approximately 250 ng of total RNA was prepared for sequencing using the rRNA depletion – Illumina Truseq stranded RNA LT kit RiboZero. The final average library size distribution was approximately 347 base pairs. Each library was spliced to a unique adapter, and they were equimolarly distributed over four lanes of an Illumina flow cell. A 100 base pairs paired-end run was performed through Genomics Facility at the University of Bristol on an Illumina HiSeq 2500 platform. The data were processed using RTA version 1.18.61 with default filter and quality settings. The reads were demultiplexed with CASAVA 1.8.2 (allowing no mismatch).

### RNA-Seq data processing

Raw data were analysed in part via public Galaxy servers (www.usegalaxy.org).^17,18^ Data sets were concatenated head to tail and processed using Trimmomatic v0.32.^19^ They were converted to Sanger format using FASTQ Groomer.^20^ Reads were then aligned to University of California, Santa Cruz (USCS) rn5 rat genome using TopHat v2.0.9^21^ default settings with the following exceptions: the maximum edit distance was set to four base pairs and four final mismatches were allowed. Coverage search and microexon search were also allowed. TopHat output was then assembled using Cufflinks v2.2.1^22,23^ with default settings and enabled bias correction to USCS rn5 genome. Data were then fed to Cuffquant and Cuffdiff^22,23^ quantitation pipeline with default setting with active multi-read-correction and bias correction options, and using the per-condition dispersion estimation method.

### RNA-Seq data analysis

Data processing was performed in R^24^ v3.2.0 using CummeRbund package v2.7.2, Qiagen Ingenuity Pathway Analysis (IPA – release June 2015) and Panther database.^25^ Gene heat-maps were created using GENE-E version 3.0.204.^26^ Benjamini–Hochberg (BH) corrected *p* values less than 0.05 were considered statistically significant. Additionally, genes had to show (at least) in one condition (WKY or SHR) an FPKM value of equal or greater than 1. Changes in genes where both conditions had FPKM < 1 were considered to be noise related and discarded from this analysis despite being statistically significant.

### Histology, image acquisition and analysis

Brain tissue was collected by decapitation and fixed in 4% PFA as previously described.^10^ After paraffin embedding, sections were cut on a microtome and stained with either picrosirius red (10 µm thick) or orcein (5 µm thick) according to standard protocols.^27^

Picrosirius red stained vessels were imaged on a Leica SP5 confocal microscope with a 40 × /0.7 oil/glycerol immersion lens as previously described.^28^ The Z stack was then maximally projected in Fiji and digital masks were applied to tunica externa and media using a semi-automatic macro. The image was then processed as described in online supplemental methods and fluorescence and area of the masks measured.

Second-harmonic generation (SHG) microscopy was performed on an upright SP8 microscope (Leica Microsystems) equipped with a DeepSee Ti:Sapphire multiphoton laser (Spectra Physics) to analyse changes in collagens. Unstained sections measuring 10 µm were imaged. The multiphoton laser was tuned to 880 nm to produce an SHG signal from collagen within the tissue samples. The signal was captured in the forward (mature collagen fibres) and backward (immature collagen fibres) direction simultaneously onto non-descanned PMTs through matched 440/20 nm emission filters. Prior to acquisition, the condenser was aligned following standard Köhler illumination and laser powers, and detector gains remained constant throughout the duration of the study.

For fluorescence and SHG microscopy, the images were processed as described in the online supplemental methods and fluorescence and area of the region of interests measured.

The orcein stained sections of basilar arteries were imaged on an upright Leica DM LB2 with a Colour Leica DCF 450C camera and 63 × dry lens. The elastin content was assessed by measurement of internal elastic lamina (IEL) area using the magic wand function in Fiji.

### Statistical analysis

Statistical analysis was performed in GraphPad Prism version 6.05. Histological samples were blinded to the experimenter prior data analysis. Provided the requirements were satisfied, an unpaired Student’s *t*-test was used for measurements of histological data. Otherwise, a non-parametric Mann–Whitney test was used. The effect size was estimated using Cohen’s *d* index value and correlation by *r* index. We considered *p* values less than 0.05 as statistically significant.

## Results

### Gene transcription shift in the SHR cerebral arteries

Gross visualisation of changes in gene transcription was achieved by hierarchical clustering. Longitudinal gene changes of WKY rats were compared to SHR. The transcriptomic changes between WKY rats at 5 and 9 weeks old were examined (genes that FDR corrected *p* value was <0.05). These genes were then extrapolated longitudinally to 13-week-old WKY rats and changes were compared in the SHR. Unchanged genes were not considered in this analysis.

As WKY rats aged, there were two groups of gene changes – those that were either up- or down-regulated in aged 9-/13-week-old WKY rats compared to 5-week-old counterparts (Figure 1(C)).

#### Upregulated genes

Three different broad clusters emerged from the upregulated genes (Figure 1(C, a to c)). The first cluster (Figure 1(C, a)) showed 85 genes that were silent in 5 relative to 9-week-old WKY rats and become silenced again at 13 weeks old. In contrast, in the SHR these genes were relatively silent in 5- and 9-week-old animals and became relatively over-transcribed at 13 weeks of age.

The second cluster (Figure 1(C, b)) contained 55 genes. They were downregulated in the 5-week-old WKY rats and became progressively upregulated in 9- and 13-week-old WKY rats. The SHR followed the same pattern of expression.

The third cluster (86 genes) showed genes that were downregulated in 5- and 9-week-old WKY rats but became strongly transcribed at 13 weeks. In the SHR, these genes did not show a consistent pattern: some remained relatively downregulated at all ages while others were over-transcribed but not to the same extent as age-matched WKY rats.

#### Downregulated genes

For downregulated longitudinal mRNA changes only two large clusters were identified (Figure 1(C, d and e)). One cluster (Figure 1(C, d) – containing 92 genes) showed a high level of downregulation at 9 weeks and a moderate upregulation at 13 weeks of age in WKY rats. In the SHR, these genes were progressively downregulated at both 9 and 13 weeks of age.

The second cluster ((Figure 1(C, e))) containing 196 genes showed progressive downregulation in the WKY rat. A similar pattern of expression was also evident in the SHR.

**Figure 2.**
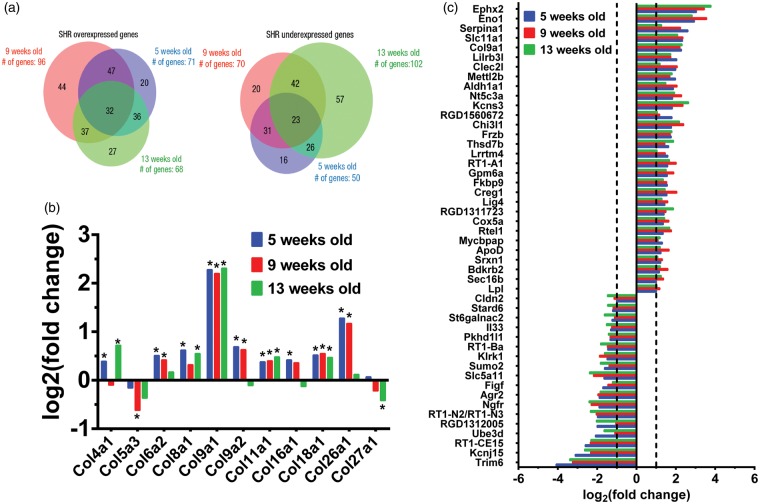
Representation of genes that showed log_2_(fold change) ≥ 1 and log_2_(fold change) ≤ 1 at various stages of hypertension development in SHR. Genes were divided into over- and under-expressed relative to WKY rats for the three ages studied (5, 9 and 13 weeks old). (a) The Venn diagrams show the number of genes unique and common to various ages and those that are constitutively changed at all ages. The sizes of the circles are proportional to total number of genes. (b) Genes constitutively upregulated and downregulated, at all analysed time points in the SHR as compared to WKY rat with log_2_(fold change) ≥ 1 and log_2_(fold change) ≤ 1. (c) Various collagens that showed differential expression in the SHR as compared to WKY. Agr2: anterior gradient protein 2 homolog; Aldh1a1: aldehyde dehydrogenase 1 family, member A1; ApoD: apolipoprotein D; Bdkrb2: Bradykinin receptor B2; Chi3l1: Chitinase-3-like protein 1; Cldn2: Claudin-2; Clec2l: C-type lectin domain family 2 member L; Col9a1: collagen type IX alpha 1 chain; Cox5a: cytochrome c oxidase subunit 5A, Creg1: cellular repressor of E1A stimulated genes 1; Eno1: Enolase-1; Ephx2: epoxide hydrolase-2; Figf: C-fos-induced growth factor; Fkbp9: FK506 binding protein 9, Frzb: frizzled-related protein; Gpm6a: Glycoprotein M6A; Il33, interleukin-33; Kcnj15, potassium inwardly-rectifying channel, subfamily J, member 15; Kcns3, potassium voltage-gated channel modifier subfamily S member 3; Klrk1, killer cell lectin like receptor K1; Lig4: DNA Ligase 4; Lilrb3l, leukocyte immunoglobulin-like receptor, subfamily B, member 3-like; Lpl, lipoprotein lipase; Lrrtm4, leucine rich repeat transmembrane neuronal 4; Mettl2b, methyltransferase like 2B; Mycbpap, MYCBP associated protein; Ngfr, nerve growth factor receptor; Nt5c3a: cytosolic 5′-nucleotidase 3; Pkhd1l1: polycystic kidney and hepatic disease-like 1; RGD1311723: centrosomal protein 295; RGD1312005: similar to DD1; RGD1560672: coiled-coil domain containing 190; RT1-A1: RT1 class Ia, locus A1; RT1-Ba: RT1 class II, locus Ba; RT1-CE15: RT1 class I, locus CE15; RT1-N2/RT1-N3: RT1 class Ib, locus N2/RT1 class Ib, locus N3; Rtel1: regulator of telomere elongation helicase 1; Sec16b: Sec16 homolog B, Serpina1: Alpha-1-antitrypsin; Slc11a1: natural resistance-associated macrophage protein 1; Slc5a11: sodium/myo-inositol cotransporter 2; Srxn1: sulfiredoxin 1; St6galnac2: sialyltransferase 7B; Stard6: StAR-related lipid transfer protein 6; Sumo2: small ubiquitin-related modifier 2; Thsd7b: thrombospondin type 1 domain containing 7B; Trim6: tripartite motif containing 6; Ube3d: ubiquitin protein ligase E3D.

Of all differentially transcribed genes, only a few genes showed constitutive, >twofold change across all ages analysed (Figure 2(a)). Of these, 32 genes were constitutively over-transcribed and 23 constitutively under-transcribed in the SHR (Figure 2(b)). There were also several collagen types that were changed (Figure 2(c)).

#### Classifying gene changes

All the differentially expressed genes were assigned to one of 28 protein classes using the Panther database (online Supplementary Figure S1). The most under- or over-transcribed class of genes were receptors with 80, 102 and 112 differentially expressed genes for 5-, 9- and 13-week-old animals, respectively. The receptor type changed the most was G-protein coupled receptors (16.5%, 17.7% and 20% of the total number of receptor genes for 5-, 9- and 13-week-old animals, respectively). The next category was hydrolases followed by enzyme modulators, nucleic acid binding proteins and transporters.

Based on the molecular function of these genes (online Supplementary Figure S1) the highest number of differentially expressed genes were enzymes (148, 242 and 260 genes for 5-, 9- and 13-week-old animals, respectively) followed by genes that show binding activity (145, 239 and 247 genes for 5-, 9- and 13-week-old animals, respectively) and receptor activity (78, 100 and 107 genes for 5-, 9- and 13-week-old animals, respectively).

IPA software identified a number of processes that these differentially expressed genes may be involved in. Based on gene set enrichment, this analysis assigns gene sets to predefined networks but no directionality is assigned, that is it cannot be stated whether these are up- or downregulated.^29^

There were 20, 69 and 18 processes (for 5-, 9- and 13-week-old animals B-H *p* value < 0.05, respectively) that these differentially expressed genes may be involved in (online Supplementary Figure S2). Of these, 15 processes were common in all three age groups (online Supplementary Table S1).

**Figure 3.**
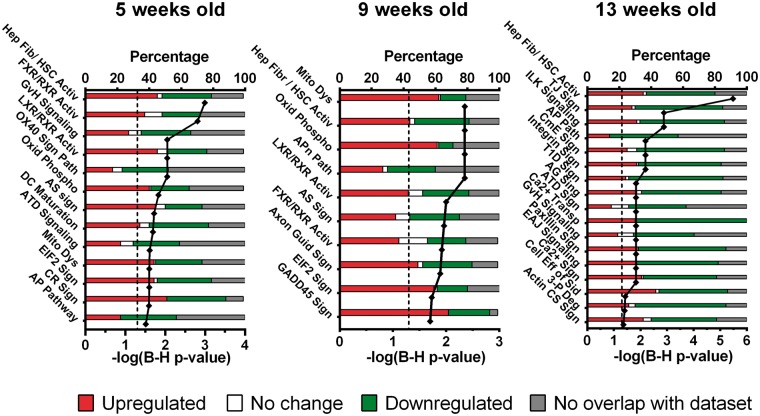
Global canonical pathways that may play a role in cerebrovascular remodelling in the SHR at various stages of hypertension development. The pathways were derived from transcriptomic changes between age-matched SHR and WKY rats and sorted according to the likelihood of involvement in the SHR cerebral artery phenotype such as immune system in the pre-hypertensive SHR. At the onset of the hypertension the stress signalling genes (e.g. GADD45) is upregulated and only after hypertension is established does endothelial dysfunction occur. This indicates that vascular remodelling at the pre-hypertensive stage is different to that during established hypertension. The onset of hypertension triggers an intermediate phenotype, presumably as a response to the increasing blood pressure. The *p* values are corrected for false discovery rate (Benjamini–Hochberg correction). The stacked bar columns represent the percentage of genes involved in a particular signalling pathway and their expression level compared to age-matched control rats (WKY). Hep Fib/ HSC Activ: hepatic fibrosis / hepatic stellate cell activation; FXR/RXR Activ: FXR/RXR activation; GvH: Graft-versus-Host disease; LXR/RXR Activ: LXR/RXR activation, OX40 Sign Path: OX40 signalling pathway; Oxid Phospho: oxidative phosphorylation; AS: atherosclerosis signalling; DC Maturation: dendritic cell maturation; ATD Signalling: autoimmune thyroid disease signalling; Mito Dys: mitochondrial dysfunction, EIF2 Sign: EIF2 signalling; CR sign: circadian rhythm signalling; AP Pathway: antigen presentation pathway, Axon Guid Sign: axonal guidance signalling; GADD45 Sign: GADD45 signalling; TJ Sign: tight junction signalling; ILK: integrin linked kinase; CmE: clathrin-mediated endocytosis signalling; T1D sign: type I diabetes mellitus signalling; AG Sign: axonal guidance signalling; Ca2+ Transp: calcium transport; EAJ: epithelial adherens junction; Ca2+ Sign: calcium signalling; Cell Eff of Sid: cellular effects of sildenafil; 3-P Deg: 3-phosphoinositide degradation; Actin CS Sign: actin cytoskeleton signalling.

There were 16, 10 and 17 pathways that were enriched statistically (B-H *p* value < 0.05) in the 5-, 9- and 13-week-old SHR, respectively (Figure 3, online Supplementary Table S1). Only two were highlighted at all three ages – hepatic fibrosis (*p* = 0.001, *p* = 0.004 and *p* = 0.000003 for 5-, 9- and 13-week-old rats, respectively) and an antigen presentation pathway (*p* = 0.03, *p* = 0.004 and *p* = 0.007 for 5-, 9- and 13-week-old animals, respectively). Of the pathways involved in maturation of the blood vessels in hypertension, six were specific to pre-hypertensive, 5-week-old SHR. During the development of hypertension (9-week-old SHR) only one pathway was specific to that stage (GADD45 pathway) while 12 pathways were specific to 13-week-old SHR, a time when hypertension is established. In general, the pathways affected most in the pre-hypertensive stage were heavily involved in immune system modulation (e.g. Graft-vs-Host signalling, OX40 signalling, atherosclerosis signalling, dendritic cell maturation, autoimmune thyroid disease signalling and antigen presentation pathways, online Supplementary Tables S2 and S3) as well as lipid and cholesterol metabolism (FXR/RXR activation and LXR/RXR activation). Processes involved in mitochondrial dysfunction and ATP production (oxidative phosphorylation) were also evident.

At the development of hypertension, two additional pathways were affected – GADD45 signalling and axon guidance signalling but Graft-vs-Host disease signalling, OX40 signalling and dendritic cell maturation signalling, remained unchanged (*p* = 0.1, *p* = 0.06 and *p* = 0.2, respectively). After hypertension was established the gene changes were statistically linked to additional pathways including tight junction signalling, integrin signalling, epithelial adherens junction signalling and cellular effects of Sildenafil suggestive of change in blood–brain barrier function.

### Putative upstream activation of differential gene expression

**Figure 4.**
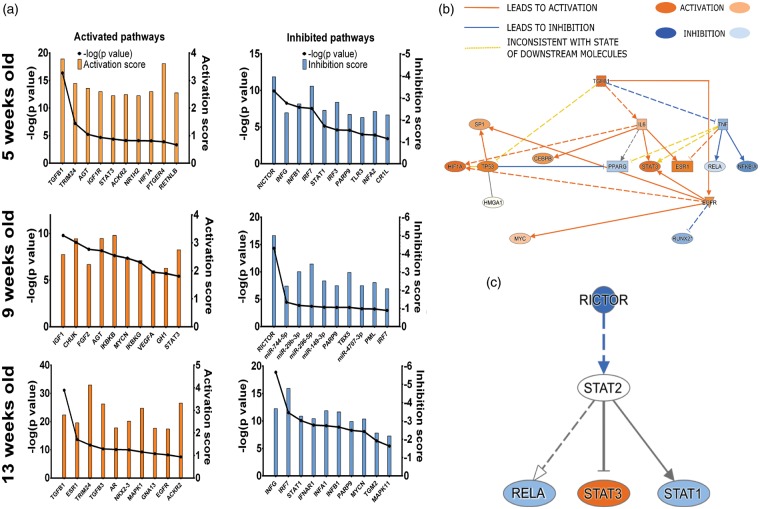
Changes in predicted upstream regulators of transcriptomic changes in the cerebral arteries of the SHR. (a) The top 10 activated and inhibited regulators across all ages of analysed samples that may potentially be involved in the age-related transcriptomic changes in the cerebral vasculature in the SHR are shown. The interplay of (b) activation of pro-fibrotic and (c) inhibition of vasoprotective regulators are also shown as inferred by IPA analysis. Pro-fibrotic regulators are inferred to be activated whereas the vasoprotective regulators are inhibited early in the pre-hypertensive SHR. The activation of pro-fibrotic regulators persists throughout all analysed time points. Additionally, at the onset (9 weeks) of hypertension, there is inhibition of miRNAs that are thought to be anti-proliferative and anti-migratory, suggestive of increased proliferation, likely of vascular smooth muscle cells – hallmark of vascular remodelling in hypertension. p-Values are reported as exact Fischer values. The relationship edges are coloured orange if they lead to activation and blue if they lead to inhibition of a downstream node. The edge is yellow when the relationship cannot be explained by the state of differential gene transcription. ACKR2: atypical chemokine receptor 2; AGT: angiotensiongen; AR: androgen receptor; CEBPB: CCAAT/enhancer-binding protein beta; CHUK: conserved helix-loop-helix ubiquitous kinase; CR1L: complement component receptor 1-like protein; EGFR: epidermal growth factor receptor; ESR1: oestrogen receptor; FGF2: fibroblast growth factor 2; GH1: somatotropin; GNA13: guanine nucleotide-binding protein subunit alpha-13; HIF1A: hypoxia-inducible factor 1-alpha; HMGA1: high mobility group protein HMG-I/HMG-Y; IGF1: insulin-like growth factor 1; IGFR1: insulin-like growth factor 1 receptor; IKBKB: inhibitor of nuclear factor kappa-B kinase subunit beta; IKBKG: NF-kappa-B essential modulator; IL6: interleukin-6; INFA1: interferon alpha-1; INFA2: interferon alpha-2; INFAR1: interferon-α/β receptor; INFB1: interferon beta-1; INFG: interferon gamma; IRF3: interferon regulatory factor 3; IRF7: interferon regulatory factor 7; MAPK1: mitogen-activated protein kinase 1; MAPK11: mitogen-activated protein kinase 11; MYCN: N-myc proto-oncogene protein; NFKBIA: NF-kappa-B inhibitor alpha; NKX2-3: NK2 transcription factor related, locus 3; NR1H2: oxysterols receptor LXR-beta; PARP9: poly [ADP-ribose] polymerase 9; PML: promyelocytic leukaemia protein; PPARG: peroxisome proliferator-activated receptor gamma; PTGER4: prostaglandin E2 receptor EP4 subtype; RELA: REL-associated protein; RICTOR: Rapamycin insensitive companion of mTOR; RUNX2: Runt-related transcription factor 2; SP1: transcription factor Sp1; STAT1: signal transducer and activator of transcription 1; STAT3: signal transducer and activator of transcription 3; TBX5: T-box transcription factor 5; TGFB1: transforming growth factor beta-1; TGFB3: transforming growth factor beta-1; TGM2: glutamine-gamma glutamyltransferase 2; TLR3: toll-like receptor 3; TNF: tumour necrosis factor; TP53: tumour protein p53; TRIM24: tripartite motif-containing 24; VEGFA: vascular endothelial growth factor A.

We analysed putative upstream regulators driving differential transcriptomic changes. The regulator itself does not have to change but drives phenotypic changes.^30^ The inhibition and activation score of these molecules is calculated in IPA. Inhibition/activation *Z*-score <2 or >2 is considered highly inhibited or activated, respectively. The top 10 activated and inhibited regulatory molecules for each age were analysed and presented in Figure 4(a). As in the canonical pathway analysis (see above), there was a progressive phenotypic shift from the pre-hypertensive, through to the onset and into the established hypertensive phase.

#### Activated regulators

At the pre-hypertensive age, the vascular transcriptomic gene changes were predicted to be caused by pro-fibrotic regulators (e.g. transforming growth factor beta 1 – TGFB1, *p* = 4 × 10^−17^, signal transducer and activator of transcription 3 – STAT3, *p* = 7.8 × 10^−5^, hypoxia inducible factor 1 alpha – HIF1A, *p* = 1.35 × 10^−4^) and regulators involved in immune system modulation (e.g. atypical chemokine receptor 2 – ACKR2, *p* = 8.13 × 10^−5^, nuclear receptor subfamily 1, group H, member 2 – NR1H2, *p* = 8.13 × 10^−5^, STAT3).

At the onset of hypertension, a combination of growth factors (e.g. insulin-like growth factor 1 – IGF1, basic fibroblast growth factor – FGF2, vascular endothelial growth factor A – VEGFA, somatotropin – GH1) and nuclear factor receptor kappa (NF-κB) signalling modulation was inferred (e.g. inhibitor of nuclear factor kappa-B kinase subunit beta and gamma – IKBKB and IKBKG, nuclear factor NF-kappa-B inhibitor kinase alpha – CHUK).

In the established hypertension phase, a partial reversal to the pre-hypertensive pro-fibrotic and immune modulation dysfunction was inferred. Additionally, the transcriptomic changes in this phase of hypertension appear to be driven by sex hormone-related signalling axis (e.g. oestrogen receptor alpha – ESR1, androgen receptor – AR). An example of the interplay between activated regulators is shown in Figure 4(b).

#### Inhibited regulators

At the pre-hypertensive age, the transcriptomic changes were largely driven by inhibition of toll-like receptor 3 (TLR3) signalling axis (interferon regulatory factor 3 – IRF3) and type I interferon signalling axis (e.g. interferon alpha 1 and beta 1 – IFNA1 and IFNB1, interferon regulatory factor 7 – IRF7, signal transducer and activator of transcription 1 – STAT1). The possible role of Rapamycin-insensitive companion of mTOR (RICTOR) in this signalling axis is highlighted in Figure 4(c). Additionally, dysregulation of complement was inferred by inhibition of complement component 3b/4b receptor 1-like (CR1L).

At the onset of hypertension several microRNAs appeared to have been inhibited (e.g. miR29b-3p, miR744-5p). The inhibition of RICTOR and IRF7 signalling persisted through this stage of hypertension development.

After hypertension becomes established the inhibition of signalling axes from the pre-hypertensive stage becomes apparent once more (e.g. inhibition of IFNA1/IFNB1-IRF7-STAT1).

### Fibrosis points towards changes in extracellular matrix deposition in pre-hypertensive rats; proof of concept

**Figure 5.**
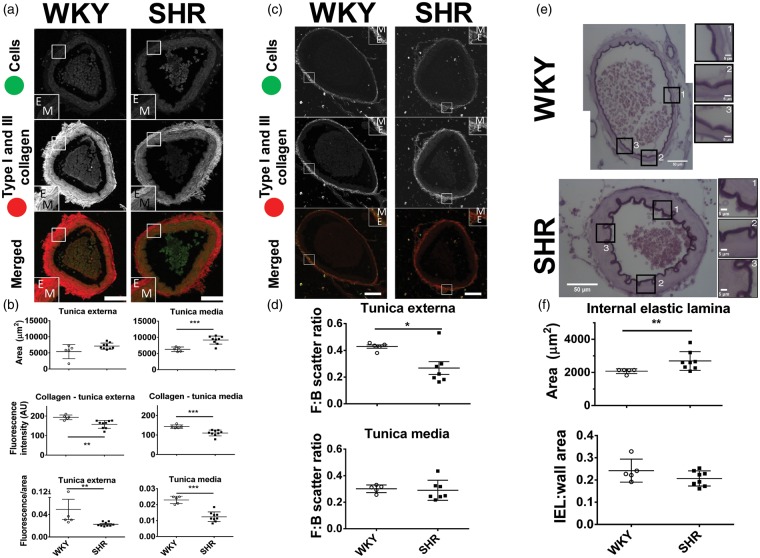
Comparison of the fibrillar collagen content in basilar arteries in 5-week-old SHR and WKY. (a) Representative images of the basilar arteries. Inset boxes indicate high power images of a section through the artery wall. (b) Tunica externa had the same area in both strains, but the area of tunica media was larger in the SHR. The total collagen (type I and III) fluorescence was lower in both tunica externa and media in the SHR relative to WKY rats. Collagen fluorescence to area ratio was reduced in the SHR compared to WKY rat in both tunica externa and media. (c) Representative images of basilar arteries. (d) Forward to backward (F:B) scatter ratios of tunica externa and media. The decreased F:B ratio in tunica externa of SHR suggests an increased level of fibrillogenesis of collagen type I. This in conjunction with decreased total levels of fibrillar collagen suggests an increase of collagen type I to type III ratio. (e) Representative images of basilar arteries stained with orcein to visualise internal elastic lamina (IEL). (f) The IEL area was greater in the pre-hypertensive SHR as compared to age-matched WKY rat. The IEL:wall area remained constant, and there was a shift in collagen type I to collagen III ratio. Scale bar = 50 µm. Values are mean ± SD. *p < 0.05, **p < 0.01, ***p < 0.001. E: tunica externa; M: tunica media.

The fibrosis pathway was a common denominator of the progression of the remodelling as the SHR ages (Figure 3). Based on this, we hypothesised that activated fibrotic pathways increased collagen content in the basilar arteries in the pre-hypertensive stage, contributing to their stiffness and reduced compliance. However, contrary to our hypothesis, combined collagen I and III content (visualised by picrosirius red fluorescence) was diminished in pre-hypertensive basilar arteries of the SHR (Figure 5(a) and (b)). The collagen fluorescence of tunica externa of the 5-week-old SHR was 19% lower than that of age-matched WKY rats (157.6 ± 20.2 vs. 193.7 ± 12.0 AU (arbitrary units), respectively, *p* < 0.01, *d* = 2.17, *r* = 0.74). Tunica media also showed lower fluorescence intensity of collagens I and III in the SHR as compared to age-matched WKY rat (23% reduction, 110.1 ± 15.0 vs. 142.9 ± 8.8 AU, *p* < 0.001, *d* = 2.67, *r* = 0.80). Comparatively the ratio of fluorescence to the area of tunica externa and media were lower in the SHR (*p* < 0.01 and *p* < 0.001, respectively). However, consistent with our hypothesis, mRNA levels of several other collagen types subunits (e.g. col4a1, col9a1) were all increased in SHRs relative to age-matched WKY (Figure 2(c)).

As picrosirius red stains all fibrillar collagen (in vessels mainly collagen type I and III) it was not clear which type was diminished in the pre-hypertensive SHR. To further explore this, we performed second harmonics generation (SHG) microscopy on these vessels. The SHG provides information based on light scattering that passes through the structures (in vessels collagen type I). Light scattered in the forward direction reflected mature collagen fibrils whereas in the backward direction immature collagen fibrils. Therefore, by comparing the forward to backward scatter (F:B ratio) we compared relative collagen type I synthesis between conditions and deduced the fibrillogenesis state.

There was an increase in the forward scatter signal in both tunica externa and media of the basilar arteries in pre-hypertensive SHR (19.19 ± 5.54 vs. 11.06 ± 3.42 AU, *p* < 0.05, *d* = 1.77, *r* = 0.66 and 5.33 ± 0.73 vs. 3.82 ± 0.36 AU, *p* < 0.01, *d* = 2.62, *r* = 0.80, respectively) as compared to age-matched WKY rat (Figure 5(c), Table 1). The backward scatter signal was also increased in the basilar arteries of pre-hypertensive SHR when compared to age-matched WKY rats (25.19 ± 8.55 vs. 87.01 ± 42.80 AU, *p* < 0.05, *d* = 2.00, *r* = 0.71). Intriguingly, the F:B ratio in the tunica externa was lowered in the pre-hypertensive SHR as compared to age-matched WKY rat (Figure 5(d) and Table 1, 0.26 ± 0.13 vs. 0.43 ± 0.03, *p* < 0.05, *d* = 1.75, 0.66) but not in the tunica media (0.29 ± 0.08 vs. 0.30 ± 0.03, *p* = 0.76, *d* = 0.20, *r* = 0.10). This suggests increased collagen type I synthesis/turnover in the adventitia of the basilar arteries of the SHR.

**Table 1.** Comparison of forward and backward scatter of 5 weeks old basilar arteries from the SHR and WKY rat.

	WKY (*n* = 5)	SHR (*n* = 7)	*p* Value	Cohen’s *d*
Tunica externa forward (F) scatter (AU)	11.06 ± 3.42	19.19 ± 5.54	0.024	1.76
Tunica media forward scatter (AU)	3.82 ± 0.36	5.33 ± 0.73	0.003	2.62
Tunica externa backward (B) scatter (AU)	25.19 ± 8.55	87.01 ± 42.80	0.012	2.00
Tunica media backward scatter (AU)	12.87 ± 2.43	19.80 ± 6.19	0.055	1.47
Tunica externa, F:B ratio	0.43 ± 0.03	0.26 ± 0.13	0.02	1.83
Tunica media, F:B ratio	0.30 ± 0.03	0.29 ± 0.08	0.76	0.20

Note. Second-harmonic generation microscopy was performed to assess collagen type I content within sections of basilar artery. The signal was captured in the forward and backward direction simultaneously. The increase in forward scatter signal of both tunica externa and media of the SHR basilar artery suggest an increase of fibrillar collagen type I. The increase of backward scatter in the tunica externa in the basilar artery of the SHR suggests increased fibrillogenesis indicating higher synthesis turnover of collagen type I. Values are mean ± SD.

The IEL – containing elastic fibres had a greater area in the 5-week-old SHR than in an aged matched WKY (2692 ± 565 vs. 2076 ± 142 µm^2^, *p* < 0.05, *d* = 1.50, *r* = 0.60, Figure 5(e) and (f)). This was consistent with upregulation of elastin and fibrillin 2 transcripts at pre-hypertensive stage (online Supplementary Figure S4). The ratio of IEL to media area, however, remained constant.

## Discussion

This study was conducted to identify global gene expression changes in the main posterior cerebral arteries prior to, and during, the development of hypertension in the SHR. The transcriptomic changes were most consistently linked to the fibrosis and immune system regulatory pathways. These in turn might have been driven by pro-fibrotic (e.g. TGFβ), anti-apoptotic (e.g. IGF1) and pro-migratory (e.g. FGF2) molecules as they were all activated in the pre-hypertensive SHR. Immune modulation signalling (e.g. interferons, TLR3 and STAT1 signalling) all appeared to be inhibited at all ages in the SHR compared to the WKY rat. A surprising finding was the plasticity of the transcriptomic changes. It appeared that the remodelling of the cerebral arteries in the pre-hypertensive SHR is different phenotypically to both the onset and developed hypertension phases of this rat model. For example, endothelial dysfunction was not apparent until hypertension had developed as shown by the enrichment of endothelial-specific pathways (e.g. tight junction signalling). Moreover, relatively few genes were persistently over- and under-transcribed at all analysed time points. Below we discuss how the alterations in transcriptomic expression may contribute to the changes in collagen content and stiffening of the cerebral vessels in the SHR, which support our selfish brain hypothesis of hypertension.

### Limitations

Several limitations must be acknowledged in the current study. It was performed on whole vessels without discrimination to the respective cell types within the vessel itself. Therefore, some pathways cannot be assigned to specific cell type, for example proliferation can refer to immune system cells, smooth muscle, fibroblasts and/or endothelium. The database exploration and modelling are based on current knowledge and available literature, and so many uncharacterised genes have been omitted from our analysis.

### Mechanisms of arterial stiffening in the SHR

As visualised by picrosirius red staining, total collagen was decreased in the basilar arteries of 5-week-old SHRs. Total fibrillar collagen (type I and III) was reduced by approximately 20% in the basilar arteries of the pre-hypertensive SHR compared to age-matched WKY rats. However, this method highlights fibrillar collagen only,^31^ which is primarily composed of collagen I, III, V in arteries^32^ but other types of fibrillar collagen exist.^33^ Although counterintuitive, a decrease in the level of fibrillar collagen in vascular remodelling is well documented. It has been proposed that the change in the ratio of collagen I and III to other types of collagen is the cause of increased arterial stiffness^34,35^ and as found herein. The decrease of fibrillar collagen we found was reported previously in 6-week-old SHRs.^35^

For the first time, we show that there is an increase in type I collagen in the basilar arteries of pre-hypertensive SHRs relative to WKY rats with discrimination to specific substructures of the blood vessels (i.e. tunica externa and media). This increased level of type I collagen may contribute to increased stiffness as it does in the heart.^36^ In the present study, SHG microscopy revealed that there was an increase in collagen I signal in both tunica externa and media in pre-hypertensive SHR. Additionally, there was a shift in forward to backward scatter (F:B) ratio of SHG signal in the tunica externa of the pre-hypertensive SHR basilar arteries suggestive of increased fibrillogenesis.^37^ Taken together, there was a shift in the collagen type I to III ratio in favour of collagen I. As collagen I provide rigidity and collagen III elasticity, the vessel with such a ratio shift will have reduced compliance, and this may contribute to the raised vascular resistance we have reported previously.^38^ We also note that there was an increase of collagen IV subunit (COL4A1). It does not escape us that mutations of this subunit may result in vascular abnormalities – in particular fragility of cerebral vessels.^39,40^ It follows then that increased expression of this subunit may conversely result in increased vessel stiffness – this notion, however, needs to be confirmed further experimentally. The vessel stiffening would have likely resulted in an increase of pulse pressure which was documented previously.^41,42^

An opinion regarding the involvement of the immune system in the generation of fibrotic tissue is that an inflammatory response occurs after vascular injury and therefore must be a secondary mechanism rather than being causal.^43^ However, our results show that an early immune response in the pre-hypertensive SHR occurs *before* the onset of hypertension and is prevailing (together with fibrosis) throughout all ages. Interestingly, one gene we identified as upregulated was alpha enolase (Eno1, Figure 2(b)) that has been linked to autoimmune disorders such as Hashimto’s encephalopathy^44^ and asthma.^45^ A symptom of one of them – Behcet disease, is a form of vasculitis. It is thought that surface alpha enolase acts as an autoantigen for endothelial damage.^46^ Given its constitutive upregulation alpha enolase may play a role in the long-term endothelial damage and immune system disorders in the SHR brainstem. Waki et al. showed an increase in adhesion of leukocytes within the brainstem microvasculature of the pre-hypertensive SHR, perhaps driving inflammation.^47,48^ Antagonism of inflammation has been successful in lowering of blood pressure in the adult SHR.^49^

The presence of immune cells in cerebral vasculature has been observed before.^48,50,51^ The early involvement of immune system pathways is suggestive of ongoing inflammation. This inflammation may be involved in production of ROS and downstream signalling that can also trigger fibrosis.^52–55^ Residing macrophages and circulating monocytes may be the source of TGFβ molecules with the ROS promoting the excessive scarring profile.^56,57^ The inhibition of interferon signalling we found may be causal to, or agonise an existing, immune response. Interferon modulates the immune system and has been used for treatment of autoimmune disorders (e.g. multiple sclerosis)^58^ as well as certain malignancies (e.g. melanoma). This remains to be validated in future work. Interestingly, a genome sequence of the SHR reveals a gain of a stop codon at amino acid position 168 (G168X) of toll like receptor 3 signalling which agrees with our model prediction of inhibition of TLR3 signalling (Figure 4).^59^

Activation of RXR signalling leads to GADD45 signalling, which has anti-proliferative properties.^60,61^ GADD45 signalling was enriched at the onset of the hypertension (9 weeks) in the SHR as well as an anti-proliferative and anti-migratory effect of miRNAs: mir-744-5p, mir-29b-3p, mir-296-5p, mir-149-3p and mir-4707-3p. Mir-744-5p has anti-proliferative properties and with their reduction increased cell proliferation may result.^62,63^ These results indicate that at the onset of hypertension there is a switch from anti- to pro-proliferative phenotype as well as increased migration, likely caused by increasing intraluminal outward pressure on the vessel wall further driving cerebral artery remodelling.

## Conclusions

All told, these findings suggest that the cerebrovascular remodelling in a rodent genetic model of hypertension shows a high degree of plasticity occurring prior to, and during the development of, hypertension. The initiating insult to the cerebral arteries is likely caused by the dysregulated pro-fibrotic and inflammatory pathways. Upon onset of hypertension, there appears to be a ‘phenotypic switch’ (increased proliferation, migration and later endothelial dysfunction) presumably to accommodate the increasing intravascular pressure.

**Figure 6.**
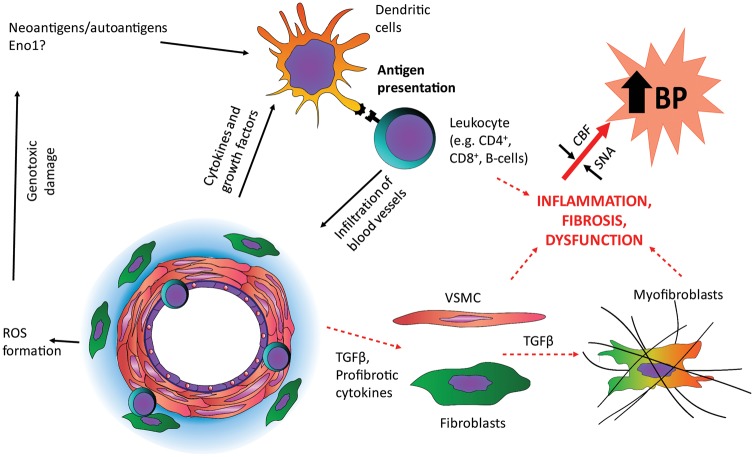
Putative pathway driving cerebrovascular remodelling in the pre-hypertensive SHR. Based on our transcriptomic analysis, we propose that some neoantigen/autoantigen (e.g. enolase-1, Eno1) drives an inflammatory pathway that leads to immune cells infiltration of blood vessels, which we have described previously.^49,51,65,66^ This causes a shift in the extracellular matrix deposition and vessel stiffening that leads to elevated cerebrovascular resistance and decreased blood flow triggering increased SNA through the Cushing mechanism. Thus, brainstem hypoperfusion results in activation of the sympathetic nervous system and hypertension. The increased blood pressure serves as a secondary insult that triggers increased vascular smooth muscle proliferation, migration and hypertrophy as well as endothelial dysfunction. These processes propagate the development of hypertension further in a positive feedback loop.

We propose a hypothetical model (Figure 6) in which a dysregulated inflammatory response leads to immune cell infiltration of cerebral blood vessels of the SHR leading to pro-fibrotic signalling that results in decreased vessel compliance and diminished cerebral blood flow at physiological blood pressure.^10^ This may lead to brainstem hypoperfusion^11^ that is counteracted by the Cushing mechanism.^15^ The increasing outward pressure on the artery wall leads to a secondary insult and phenotypic changes, worsening vessel fibrosis and compounding stiffening. The inhibition of anti-proliferative and anti-migratory miRNA effects as well as activation of growth factor signalling leads to cell proliferation and migration that result in secondary hypertrophic remodelling together with an increase in extracellular matrix turnover. As the hypertension becomes established the immune system dysfunction continues and endothelial dysfunction sets in, including a break down in blood brain barrier function.^64^ We predict that targeting of the immune system dysfunction early on in the disease process may provide an approach to prevent the development of high cerebral vascular resistance and subsequent hypertension in the SHR.

## Supplemental Material

Supplemental material for Inflammatory pathways are central to posterior cerebrovascular artery remodelling prior to the onset of congenital hypertension Click here for additional data file. Supplemental material for Inflammatory pathways are central to posterior cerebrovascular artery remodelling prior to the onset of congenital hypertension by Dawid Walas, Karol Nowicki-Osuch, Dominic Alibhai, Eva von Linstow Roloff, Jane Coghill, Christy Waterfall and Julian FR Paton in Journal of Cerebral Blood Flow & Metabolism
