# Supplementary material for: Inflammatory pathways are central to posterior cerebrovascular artery remodelling prior to the onset of congenital hypertension
Source: J Cereb Blood Flow Metab. 2018 Apr 13;39(9):1803–17. doi: 10.1177/0271678X18769180 (PMC6724458; doi:10.1177/0271678X18769180)
Supplement: Supplemental material for Inflammatory pathways are central to posterior cerebrovascular artery remodelling prior to the onset of congenital hypertension [file Supplemental_material_5.pdf]

Supplemental methods and data

Collagen content measurements

Measurements of picrosirius red staining and SHG were made using a semi-automatic macro written in ImageJ. A "middle" of each section was found. From there a series of images were taken in the Z dimension (5µm thick stack, at 0.5µm steps). A maximum intensity projection in the z dimension was applied to each stack. The user is prompted to select four different areas in each max projected image. Firstly a 'rough outline' of the vessel. Secondly, the outline of the whole vessel using the magic wand tool, the tolerance of which is adjusted manually by the user for each vessel to ensure an appropriate segmentation of the vessel occurs. Thirdly the inside of the collagen layer was segmented and lastly the lumen was segmented. The macro automatically converts these regions into binary masks and through logical AND/OR functions generates masks corresponding to:

- The outer collagen layer
- The vessel wall
- The total vessel size (including collagen and lumen)

Automatic measurements of size and shape of these masks were performed in ImageJ. The masks were also applied to the original maximum intensity image to make measurements of the picrosirius red staining. All measurements were outputted to the results/summary window in ImageJ and then further analysis.

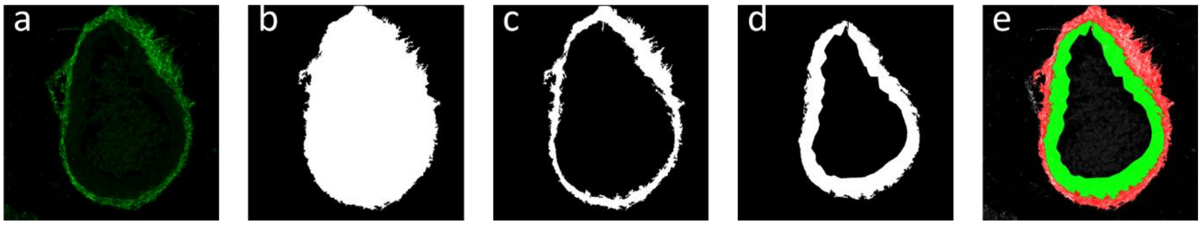

a) Maximum intensity projection of backward scattered SHG signal
b) User asked to select outer edge of vessel. Binary mask created.
c) User asked to select adventitia. Binary mask created
d) Binary mask c subtracted from binary mask b.
e) Intensity measurements made using the resultant binary masks on original data a.

Supplemental figures

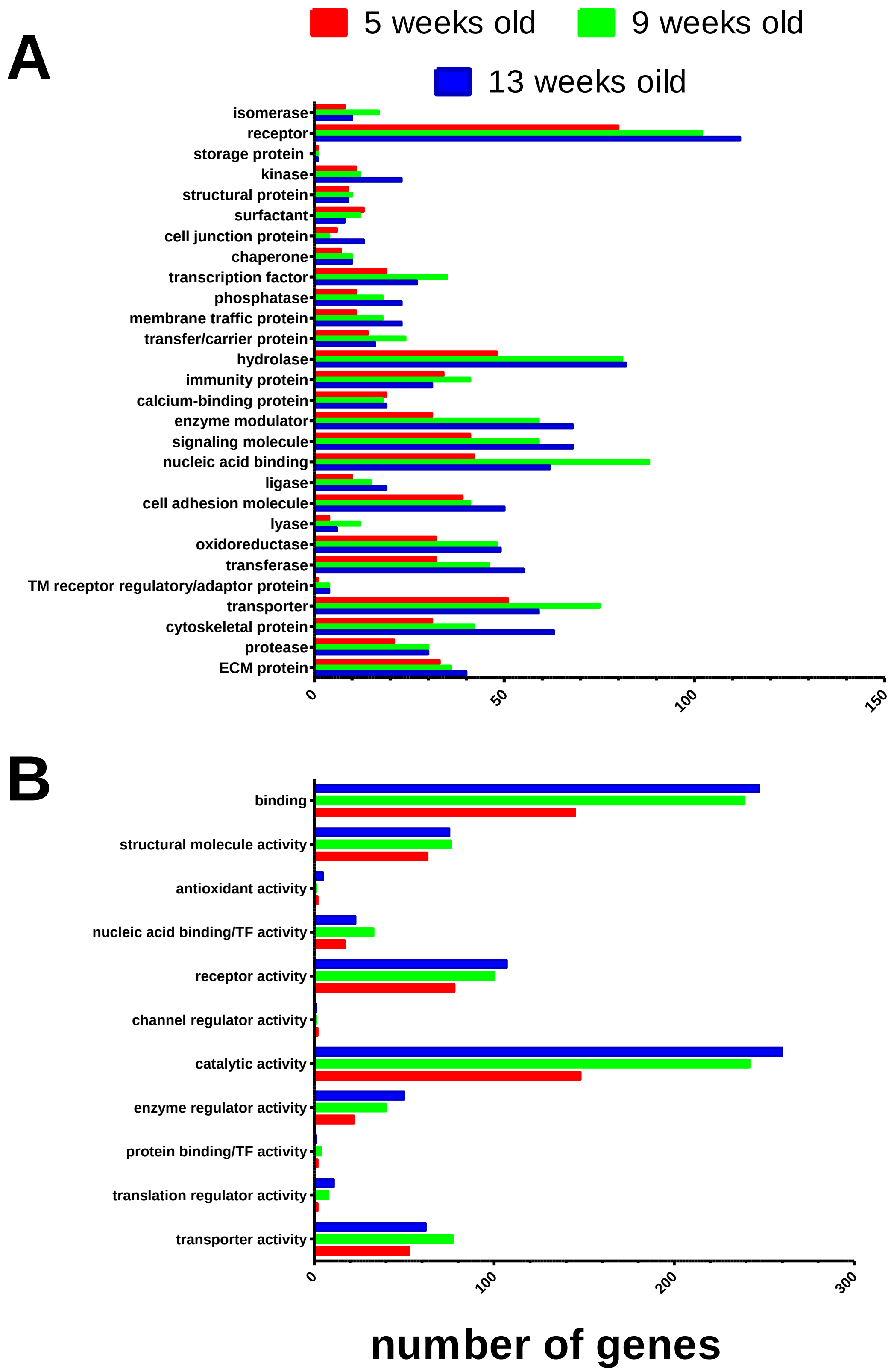

Figure S2. Panther database gene ontology analysis of differentially expressed genes

The genes presented are differentially transcribed between aged matched WKY and SHR and categorized based on their A) protein class and B) the molecular function they play.

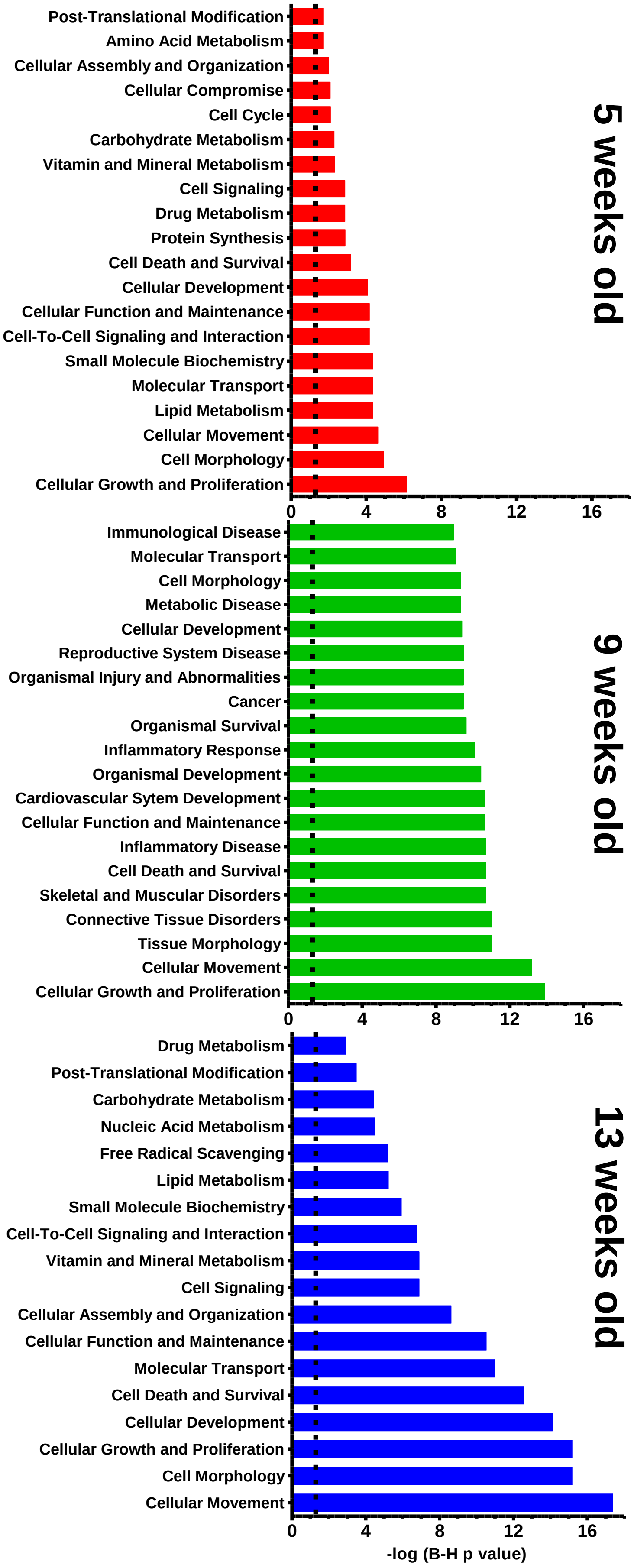
Post-Translational Modification
Amino Acid Metabolism
Cellular Assembly and Organization
Cellular Compromise
Cell Cycle
Carbohydrate Metabolism
Vitamin and Mineral Metabolism
Cell Signaling
Drug Metabolism
Protein Synthesis
Cell Death and Survival
Cellular Development
Cellular Function and Maintenance
Cell-To-Cell Signaling and Interaction
Small Molecule Biochemistry
Molecular Transport
Lipid Metabolism
Cellular Movement
Cell Morphology
Cellular Growth and Proliferation
0
4
8
12
16
5 weeks old
Immunological Disease
Molecular Transport
Cell Morphology
Metabolic Disease
Cellular Development
Reproductive System Disease
Organismal Injury and Abnormalities
Cancer
Organismal Survival
Inflammatory Response
Organismal Development
Cardiovascular Sytem Development
Cellular Function and Maintenance
Inflammatory Disease
Cell Death and Survival
Skeletal and Muscular Disorders
Connective Tissue Disorders
Tissue Morphology
Cellular Movement
Cellular Growth and Proliferation
0
4
8
12
16
9 weeks old
Drug Metabolism
Post-Translational Modification
Carbohydrate Metabolism
Nucleic Acid Metabolism
Free Radical Scavenging
Lipid Metabolism
Small Molecule Biochemistry
Cell-To-Cell Signaling and Interaction
Vitamin and Mineral Metabolism
Cell Signaling
Cellular Assembly and Organization
Cellular Function and Maintenance
Molecular Transport
Cell Death and Survival
Cellular Development
Cellular Growth and Proliferation
Cell Morphology
Cellular Movement
0
4
8
12
16
-log (B-H p value)
13 weeks old

Figure S3. Ingenuity pathway analysis of diseases and biofunctions that the differentially expressed genes might be involved in at various stage of hypertension in the SHR

A maximum of 20 biofunctions with highest probability are shown for each age. The p values reported are false discovery rate corrected (Benjamini-Hochberg correction). These biofunctions are highlighted based on pairwise comparison of differential gene expression between SHR and age matched WKY. The common biofunction across all ages analysed are listed in Table S1. The dotted line represents the threshold value of p=0.05.

	Probability [B-H corrected; –log(p value)]		
**Biofunction**	**5 weeks old**	**9 weeks old**	**13 weeks old**
**Carbohydrate Metabolism**	2.3	2.9	4.4
**Cell Death and Survival**	3.2	10.7	12.6
**Cell Morphology**	4.9	9.3	15.2
**Cell Signalling**	2.9	3.9	6.9
**Cell-To-Cell Signalling and Interaction**	4.2	6.0	6.7
**Cellular Assembly and Organization**	2.0	5.3	8.6
**Cellular Development**	4.1	9.4	14.1
**Cellular Function and Maintenance**	4.2	10.6	10.5
**Cellular Growth and Proliferation**	6.2	13.9	15.2
**Cellular Movement**	4.6	13.6	17.4
**Drug Metabolism**	2.9	2.9	2.9
**Lipid Metabolism**	4.3	4.6	5.2
**Molecular Transport**	4.3	9.1	11.0
**Small Molecule Biochemistry**	4.3	4.6	5.9
**Vitamin and Mineral Metabolism**	2.3	3.5	6.9

Table S1. Biofunctions highlighted in all age matched conditions

The probable biofunctions that are affected by differential gene expression between WKY and SHR. These biofunctions are common to all analysed ages. The probabilities reported are Benjamini-Hochberg corrected.

pathway ID	pathway description	observed gene count	false discovery rate	matching genes in the dataset
GO.0002376	immune system process	43	6.94E-07	Akt1, Anxa1, Atpif1, Bdkrb2, C1rl, C1s, C3, Ccbp2, Ccr5, Cxcr4, Cyba, Ddit4, Eef2, Eif2ak2, Emr1, Enpp3, F2rl1, Hmgb2, Irgm, Kitlg, Klrk1, Lrrc8a, Mertk, Mif, Mx1, Mx2, Narfl, Nlrx1, Oas2, Oas3, Oasl2, Olr1, Orai1, Pmaip1, Prg2, RT1-Bb, RT1-Db1, S100a9, Tcf3, Tgfb2, Thy1, Tinagl1, Vegfa
GO.0006954	inflammatory response	24	3.96E-06	Akt1, Bdkrb2, C3, Ccbp2, Ccr5, Cd24, Chi3l1, Chst1, Cxcl10, Cyba, Epha3, Ephx2, F2rl1, Gal, Hmgb2, Mif, Olr1, P2rx1, Park7, Plp1, Ptgs1, S100a9, Serpina1, Sgms1
GO.0006955	immune response	28	1.45E-05	Bdkrb2, C1rl, C1s, C3, Ccr5, Cd24, Cd4, Cxcl10, Cyba, Eif2ak2, Enpp3, F2rl1, Hmgb2, Irgm, Klrk1, Mif, Mx1, Mx2, Nlrx1, Oas2, Oas3, Oasl2, Prg2, RT1-Bb, RT1-Db1, S100a9, Tinagl1, Vegfa
GO.0002252	immune effector process	14	0.000618	C1rl, C1s, C3, Cxadr, Cxcl10, Ddit4, Eif2ak2, F2rl1, Mx1, Mx2, Oas2, Oas3, Oasl2, Pmaip1
GO.0045087	innate immune response	16	0.00117	C1rl, C1s, C3, Cyba, Eif2ak2, F2rl1, Irgm, Klrk1, Mif, Mx1, Mx2, Nlrx1, Oas2, Oas3, Oasl2, S100a9
GO.0002683	negative regulation of immune system process	13	0.00379	Adcyap1, Apod, Cd24, F2rl1, Gal, Grem1, Hist2h4, Kitlg, Mertk, RT1-Bb, Tgfb2, Tgfb3, Thy1
GO.0050727	regulation of inflammatory response	11	0.0102	Adcyap1, Agt, Apod, Ccr5, Cd24, Il33, Mif, Park7, Ptgis, S100a9, Wfdc1
GO.0002682	regulation of immune system process	21	0.0336	Adcyap1, Anxa1, Apod, C1rl, C1s, C3, Cd4, Cdc37, Eif2ak2, Gal, Grem1, Hist2h4, Kitlg, Klrk1, Mertk, Mif, RT1-Bb, Tcf3, Tgfb2, Tgfb3, Thy1
GO.0002520	immune system development	13	0.0478	Anxa1, Atpif1, C3, Cd24, Cd4, Eef2, F2rl1, Kitlg, Lrrc8a, Mertk, Narfl, Tcf3, Tgfb2

Table S2. Enrichment of GO terms associated with immune system and inflammatory responses.

Gene	Log2(Fold change)	FDR corrected p value	Information
Anxa1	-0.32	0.059	Annexin A1; Plays important roles in the innate immune response as effector of glucocorticoid-mediated responses and regulator of the inflammatory process. Has anti-inflammatory activity. Plays a role in glucocorticoid-mediated down-regulation of the early phase of the inflammatory response. Promotes resolution of inflammation and wound healing (By similarity). Functions at least in part by activating the formyl peptide receptors and downstream signalling cascades. Promotes chemotaxis of granulocytes and monocytes via activation of the formyl peptide receptors
Bdkrb2	1.16	0.002	B2 bradykinin receptor isoform 1; Receptor for bradykinin. It is associated with G proteins that activate a phosphatidylinositol-calcium second messenger system
C1rl	1.35	0.04	Complement C1r subcomponent-like protein precursor; Mediates the proteolytic cleavage of HP/haptoglobin in the endoplasmic reticulum
C3	0.83	0.004	Complement C3 precursor; C3 plays a central role in the activation of the complement system. Its processing by C3 convertase is the central reaction in both classical and alternative complement pathways. After activation C3b can bind covalently, via its reactive thioester, to cell surface carbohydrates or immune aggregates
Ccbp2	0.94	0.035	Chemokine-binding protein 2; Atypical chemokine receptor that controls chemokine levels and localization via high-affinity chemokine binding that is uncoupled from classic ligand-driven signal transduction cascades, resulting instead in chemokine sequestration, degradation, or transcytosis. Also known as interceptor (internalizing receptor) or chemokine-scavenging receptor or chemokine decoy receptor.
Ccr5	0.78	0.002	C-C chemokine receptor type 5; Receptor for a number of inflammatory CC-chemokines including MIP-1-alpha, MIP-1-beta and RANTES and subsequently transduces a signal by increasing the intracellular calcium ion level. May play a role in the control of granulocytic lineage proliferation or differentiation
Cd24	0.68	0.02	Signal transducer CD24; May have a pivotal role in cell differentiation. The triggering mechanism of signal transduction may be due to the interactions of differentiating cells with the matrix substrate via the carbohydrate structure of the molecule. In this way, the signal transducer can play very different roles in different cell types as a direct consequence of its glycosylation
Cd4	0.37	0.04	T-cell surface glycoprotein CD4; Accessory protein for MHC class-II antigen/T-cell receptor interaction. May regulate T-cell activation
Chi3l1	1.8	0.03	Chitinase-3-like protein 1; Carbohydrate-binding lectin with a preference for chitin. Has no chitinase activity. May play a role in tissue remodelling and in the capacity of cells to respond to and cope with changes in their environment. Plays

			a role in T-helper cell type 2 (Th2) inflammatory response and IL-13-induced inflammation, regulating allergen sensitization, inflammatory cell apoptosis, dendritic cell accumulation and M2 macrophage differentiation.
Cxcl10	-0.75	0.07	C-X-C motif chemokine 10 ; In addition to its role as a proinflammatory cytokine, may participate in T-cell effector function and perhaps T-cell development
Cxcr4	0.52	0.06	C-X-C chemokine receptor type 4 ; Receptor for the C-X-C chemokine CXCL12/SDF-1 that transduces a signal by increasing intracellular calcium ion levels and enhancing MAPK1/MAPK3 activation. Acts as a receptor for extracellular ubiquitin; leading to enhanced intracellular calcium ions and reduced cellular cAMP levels. Involved in haematopoiesis and in cardiac ventricular septum formation. Also plays an essential role in vascularization of the gastrointestinal tract, probably by regulating vascular branching and/or remodelling processes in endothelial cells.
F2rl1	-1.26	0.002	Proteinase-activated receptor 2 ; Receptor for trypsin and trypsin-like enzymes coupled to G proteins. Its function is mediated through the activation of several signalling pathways including phospholipase C (PLC), intracellular calcium, mitogen-activated protein kinase (MAPK), I- kappaB kinase/NF-kappaB and Rho. Can also be transactivated by cleaved F2r/Par1. Involved in modulation of inflammatory responses and regulation of innate and adaptive immunity, and acts as a sensor for proteolytic enzymes generated during infection. Generally, is promoting inflammation.
Gal	0.9	0.025	Galanin peptides Galanin Galanin message-associated peptide; Endocrine hormone of the central and peripheral nervous systems that binds and activates the G protein-coupled receptors GALR1, GALR2, and GALR3. This small neuropeptide may regulate diverse physiologic functions including contraction of smooth muscle of the gastrointestinal and genitourinary tract, growth hormone and insulin release and adrenal secretion
Grem1	0.78	0.011	Gremlin-1; Cytokine that may play an important role during carcinogenesis and metanephric kidney organogenesis, as a BMP antagonist required for early limb outgrowth and patterning in maintaining the FGF4-SHH feedback loop. Down-regulates the BMP4 signalling in a dose-dependent manner. Acts as inhibitor of monocyte chemotaxis. Can inhibit the growth or viability of normal cells but not transformed cells when is overexpressed
Il33	-1.31	0.002	Interleukin-33
Mif	0.57	0.047	Macrophage migration inhibitory factor; Pro-inflammatory cytokine. Involved in the innate immune response to bacterial pathogens. The expression of MIF at sites of inflammation suggests a role as mediator in regulating the function of macrophages in host defence. Counteracts the anti- inflammatory activity of glucocorticoids. Has phenylpyruvate tautomerase and dopachrome tautomerase activity (in vitro), but the physiological substrate is not known.
Olr1	0.87	0.028	Oxidized low-density lipoprotein receptor 1 Oxidized low-density lipoprotein receptor 1, soluble form; Receptor that mediates the recognition, internalization and degradation of oxidatively modified low density lipoprotein (oxLDL) by vascular endothelial cells. OxLDL is a marker of atherosclerosis that induces vascular endothelial cell activation and dysfunction, resulting in pro-inflammatory responses, pro- oxidative conditions and apoptosis. Its association with oxLDL induces the activation of NF-kappa-B through an increased production of intracellular reactive oxygen species

Prg2	-1.08	0.018	Bone marrow proteoglycan precursor; Cytotoxin and helminthotoxin. MBP also induces non- cytolytic histamine release from basophils. It is involved in antiparasitic defence mechanisms and immune hypersensitivity reactions
RT1-Bb	-0.62	0.004	Rano class II histocompatibility antigen, B-1 beta chain precursor; Involved in the presentation of foreign antigens to the immune system
RT1-Db1	-0.84	0.002	Rano class II histocompatibility antigen, D-1 beta chain precursor
Tgfb3	0.45	0.014	Transforming growth factor beta-3; Involved in embryogenesis and cell differentiation

Table S3. Transcripts involved in immune system signalling – receptors and ligands. Genes that transcript changed in the pre hypertensive (5 weeks old) SHR as compared to aged matched WKY

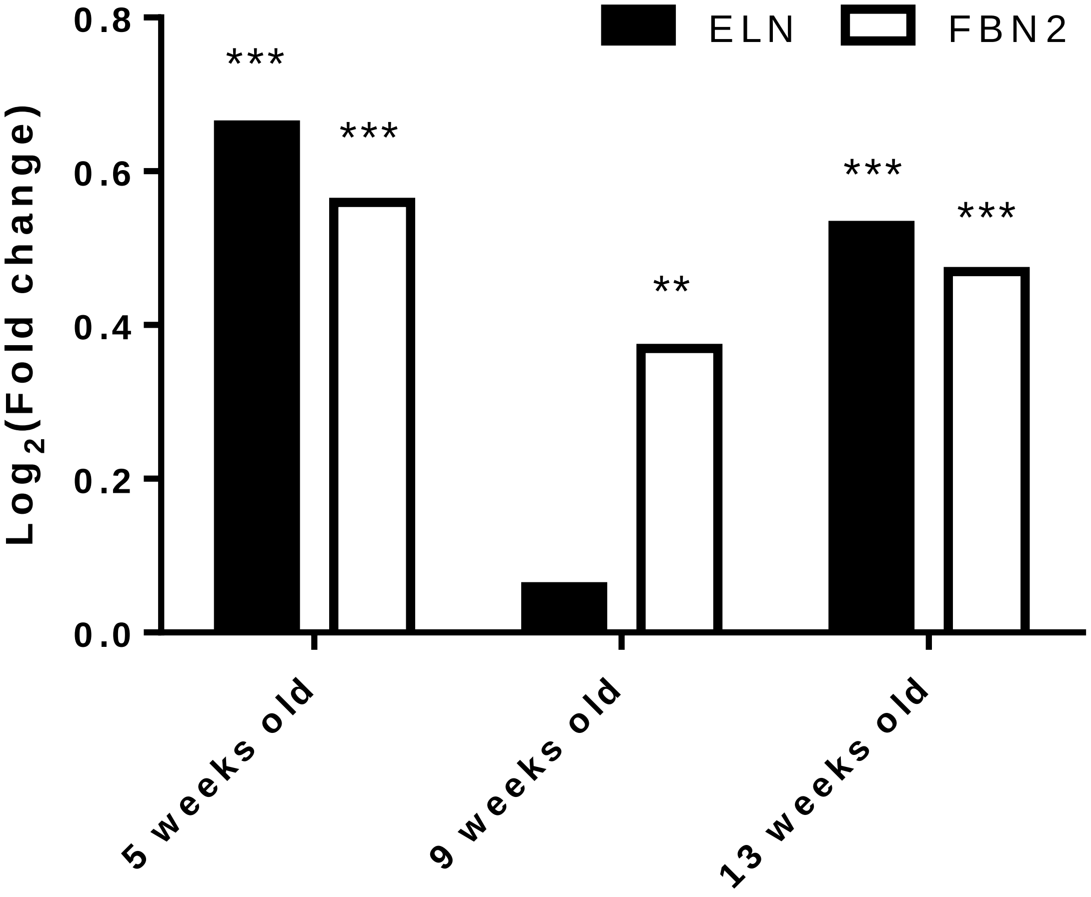

Figure S4. Elastin and Fibrillin-2 transcript level in the SHR. Elastin (ELN) and Fibrillin-2 (FBN2) trasncripts are increased in the pre-hypertensive stage of the SHR as compared to age matched WKY. *** = p<0.002, ** = p<0.01
